# The Relationship between Father–Child Rough-and-Tumble Play and Children’s Working Memory

**DOI:** 10.3390/children9070962

**Published:** 2022-06-27

**Authors:** Emily Elsa Freeman, Erin Louise Robinson

**Affiliations:** School of Psychological Sciences, The University of Newcastle, Callaghan, NSW 2308, Australia; erin.robinson@uon.edu.au

**Keywords:** rough-and-tumble play, father–child interactions, working memory, fathers, parenting

## Abstract

Rough-and-tumble play (RTP) between fathers and children has been linked to many social, emotional, and behavioural child outcomes, such as reduced aggression and increased self-regulation. This study extends our understanding of the importance of RTP to the development of the executive function, working memory. Father–child dyads (N = 30) were asked to play two RTP games that were videorecorded for later observational coding. Fathers were also asked to report the frequency with which they play RTP games with their child. Two measures of working memory were also collected. The working-memory index of the Wechsler Preschool and the Primary Scale of Intelligence—Fourth Edition were used to measure working-memory ability, and the working-memory subscale of the Behaviour Rating Inventory of Executive Function was used as a measure of working-memory problems. RTP frequency was associated with improved working-memory ability and fewer working-memory problems. RTP quality was associated with higher working-memory ability. This study adds to the growing evidence of the importance of father–child RTP for child development.

## 1. Introduction

In humans, play has been found to comprise a greater proportion of fathers’ interactions with their children compared to mothers [1,2]. Comparisons between mothers’ and fathers’ play have shown that fathers’ play is often more physical, less predictable, and more likely to include rough-and-tumble play (RTP) [3]. RTP is generally accepted as being best defined by Pellegrini and Smith [4] who described RTP as including “vigorous behaviours such as wrestling, grappling, kicking, and tumbling that would appear to be aggressive except for the playful context” (p. 579). For these reasons, studies interested in the relationship between RTP and child development have focused on father–child RTP, e.g., [3,5,6,7,8,9].

Studies investigating father–child RTP have found numerous relationships with social and emotional development. For example, Fletcher et al. [6] conducted a study with 23 father–child dyads. They found that high-quality RTP was associated with fewer Total Problems as rated by both mothers and fathers using the Strengths and Difficulties Questionnaire (SDQ) [10]. Anderson, St George, and Roggman [11] reported that RTP was negatively correlated with aggression, such that higher-quality RTP was related to lower aggression scores in a sample of 25 father–child dyads. Numerous researchers have also found links between RTP and social competence [3,6,7,12], emotion regulation [13,14], and self-regulation [6,7,8,9,11]. RTP is not unique to humans but has also been observed across numerous mammalian species [15,16,17]. Interestingly, neurological studies of rats who have been deprived of ‘play-fighting’ show deficiencies in the development of their prefrontal cortex (PFC) [15,18].

The PFC is a brain area associated with executive function [19,20]. Executive functions develop rapidly during early childhood and play a crucial role in cognitive functioning, behaviour, and social and emotional control [21,22,23,24,25]. Children’s executive function has been linked to a wide variety of outcomes including prosocial behaviour, better concentration, and self-regulation [26,27]; drug use [28,29]; physical health and obesity [29,30]; motor control [31]; theory of mind [22]; and school readiness and academic achievement [32]. Executive processes include such things as cognitive flexibility, inhibitory control, and working memory [33]. 

Working memory is a fundamental executive function that is defined as the ability to hold and manipulate information in the mind [34]. Working memory is required in almost all everyday tasks such as remembering the order of steps required to complete a task, linking letters together to form words, solving math problems, linking words together to enable comprehension, locating oneself in space, reading maps, and so forth. Working memory has been linked to numerous areas of child development including academic achievement [35,36,37] and intelligence [38,39]. Working-memory difficulties have also been associated with classroom behaviour problems [40].

Given that RTP in rats has been linked to the development of the PFC [15,17,18], and that the PFC is associated with development of executive functions [19,20], it is interesting that human studies of RTP have had such a large emphasis on social- and behavioural-outcome measures and not cognitive outcomes. Therefore, the aim of the current study was to examine the relationship between father–child RTP and children’s working-memory ability. Working memory was chosen as it has recently been shown in a meta-analysis to be the EF process with the strongest predictive power for predicting cognitive development in children [41]. Additionally, it has been suggested that EF should be measured using a combination of neuropsychological cognitive assessments and ecologically valid behavioural assessments [21]. An advantage of examining working memory as an outcome measure is that both of these cognitive and behavioural measures are available and validated for use with children. Specifically, we used the working-memory index of the Wechsler Preschool and the Primary Scale of Intelligence—Fourth Edition Australian and New Zealand Standardised Edition (WPPSI-IV) [42] as our cognitive assessment of working-memory ability. We also used the working-memory subscale of the Behaviour Rating Inventory of Executive Function (BRIEF) parent form [43] as our behavioural measure of working-memory problems. It is important to note that past research has consistently demonstrated modest agreement between the BRIEF and cognitive measures of EF, which has been interpreted as supporting the notion that it provides unique information beyond that provided by cognitive measures [21]. Based on the previous work linking RTP and the PFC in animal studies, alongside the aforementioned studies showing links between RTP and social and emotional regulation, we expected that father–child RTP frequency and quality would be associated with higher working-memory ability and fewer working-memory problems.

## 2. Materials and Methods

### 2.1. Participants

Thirty father–child dyads from a large regional city in Australia participated in this study. The fathers’ mean age was 38 years (SD = 5 years). The children (16 girls, 14 boys) were typically developing and aged between 4 and 7 years (M = 6 years, SD = 1 year). All dyads were from intact heterosexual two-parent families and reported no cognitive, developmental, or physical delays or disabilities. All fathers were employed and worked an average of 40 h per week (SD = 7 h), and 83% of the families earned over AUD 100,000 annually. This study received ethical approval from the Human Research Ethics Committee at the University of Newcastle. Fathers gave their informed consent for themselves and their child, and the study child gave their assent before the study commenced.

### 2.2. Measures

#### 2.2.1. Demographic Questionnaire

This 14-item questionnaire, completed by the father, included parent items such as age, marital status, level of education, household income, and hours of paid work each week and child items such as age, birth order, and gender of the child participating in the study.

#### 2.2.2. Rough-and-Tumble-Play Quality 

The RTP-Q [6] assesses father–child-interaction quality when engaging in RTP via observer coding. It contains 16 items, coded on a 5-point Likert Scale, that rate individual and dyadic affective states and behaviours of the father and child in terms of warmth, control, sensitivity, winning and losing, physical engagement, and playfulness. These ratings are based on both verbal and non-verbal behaviours. Scores on the RTP-Q can range from 16, indicating very poor-quality play, through to 80, indicating extremely high-quality play. The RTP-Q has been used in a variety of studies worldwide and has been found to have good reliability and validity [11,44]. Inter-item reliability in this sample was excellent, α = 0.98). Coding of the RTP using the RTP-Q was conducted by two researchers with a high level of agreement (ICC = 0.89).

#### 2.2.3. Rough-and-Tumble-Play Frequency

Fathers were asked to answer a question as to how often they participate in RTP with the study child using a 4-point scale (1 = not at all; 4 = every day). Although only a single item, this question has been used in previous studies examining the frequency of father–child RTP [5,14].

#### 2.2.4. Working-Memory Ability

The Wechsler Preschool and Primary Scale of Intelligence—Fourth Edition Australian and New Zealand Standardised Edition (WPPSI-IV) [42] was used to measure children’s working-memory ability. The WPPSI-IV is a measure of children’s cognitive ability suitable for children aged 2 years 6 months to 7 years 7 months. It consists of 15 subtests of which 2 (picture memory and zoo locations) are combined to produce the working-memory index (WMI). WMI scores are represented as standard scores with a mean of 100 and a standard deviation of 15. WMI scores have been found to be reliable across the range of age groups, with reliability coefficients ranging between 0.88 and 0.93.

#### 2.2.5. Working-Memory Problems

The Behaviour Rating Inventory of Executive Function (BRIEF) parent form [43] was used as a behavioural measure of children’s working-memory problems. The BRIEF is an assessment of executive function consisting of 86 statements relating to problem behaviours. Parents are required to respond to each statement using a 3-point scale (i.e., 0 = never, 1 = sometimes, 2 = often). The items form eight subscales covering a broad range of executive function behaviours, including working memory. The working-memory subscale is made up of 8 items and has high internal consistency (α = 0.892) [45]. The full BRIEF has good test–retest reliability for the eight subscales (r = 0.76–0.85) and high internal consistency (α = 0.80–0.98) [43]. 

### 2.3. Procedure

Father–child dyads first participated in a 10 min rough-and-tumble play session. Participants were asked to play two games during the session. Sock Wrestle required each participant to wear one sock. The aim of the game was to get their opponents sock, without losing their own. Get Up required one participant to lay on the play mat on their back and try to get up to a standing position, while their opponent tried to keep them down. While the instructions of the games were counter-balanced across participants, they were informed that they could alternate between the games as they liked for the 10 min duration. The play took place in a large room clear of toys and other distractions. The play was filmed from multiple angles via wall-mounted cameras, and the experimenter left the room during the play session. Following the RTP session, fathers were asked to complete the demographics questionnaire, the RTP-Frequency question, and the BRIEF, while the child was administered the WPPSI by one of the trained experimenters.

### 2.4. Data Analysis

Data were analysed using JASP [46], so that both frequentist and Bayesian analyses could be conducted. The use of Bayes factors is advantageous as it allows evidence in favour of the null hypothesis to be calculated. In this paper, Bayes factors are reported as values greater than one, with the subscripts indicating evidence in favour of the null, BF_01_, or alternative, BF_10_, hypothesis respectively. Additionally, Bayes factors are interpreted according to Jeffrey’s [47] descriptors, with 1–3 being anecdotal, 3–10 being substantial, 1–30 being strong, 30–100 being very strong, and >100 being decisive evidence.

## 3. Results

### 3.1. Descriptive Statistics

Table 1 outlines the descriptive statistics for each of the variables of interest separately for boys and girls. Independent-samples *t*-tests showed there was no significant difference in scores and anecdotal evidence in favour of the null hypothesis for boys and girls on the WPPSI-IV working-memory scale (t(28) = 1.47, p = 0.15, BF_01_ = 1.29), the BRIEF working-memory scale (t(28) = 0.12, *p* = 0.91, BF_01_ = 2.89), and RTP-Quality (t(28) = 0.08, *p* = 0.94, BF_01_ = 2.89). A chi-squared analysis showed no differences in RTP-Frequency for boys and girls (χ^2^ = 4.20, df = 3, *p* = 0.24, BF_01_ = 2.93). For the WPPSI-IV working-memory scale, four children had scores lower than the normal range, while for the BRIEF working-memory scale five children had elevated scores, suggestive of potential working-memory problems.

### 3.2. Relationship between Rough-and-Tumble Play and Working Memory

Correlational analyses were conducted between the RTP and working-memory measures to determine the strength and direction of the relationships. RTP-Quality was positively related to working memory as measured by the WPPSI-IV (r = 0.40, *p* = 0.037, BF_10_ = 2.34) but was not significantly correlated with the BRIEF working-memory scale (r = −0.09, *p* = 0.652, BF_01_ = 3.99). RTP-Frequency was significantly correlated with both WPPSI-IV(r = 0.53, *p* = 0.003, BF_10_ = 16.09) and BRIEF (r = −0.42, *p* = 0.020, BF_10_ = 3.06) working-memory scores. Interestingly, RTP-Quality and frequency were not significantly correlated (r = 0.12, *p* = 0.534, BF_01_ = 3.66) and neither were the WPPSI-IV and BRIEF working-memory scores (r = −0.31, *p* = 0.101, BF_01_ = 1.22).

Two stepwise linear regressions were conducted using RTP quality and frequency to predict working memory, as measured by the WPPSI-IV and BRIEF (see Table 2). Child gender was not included in these models as the descriptive analyses had not revealed any significant effects. When predicting working memory as measured by the WPPSI-IV, the final model included both RTP quality and frequency (F(2,27) = 8.83, *p* = 0.001). This model was supported by the Bayesian analysis as having the strongest support compared to the null (BF10 = 33.47) and explained 35% of the variance in WPPSI-IV working-memory scores. The best model, compared to the null, at predicting working memory as measured by the BRIEF, included only RTP frequency (F(2,27) = 6.14, *p* = 0.020, BF_10_ = 3.10). This model explained 15% of the variance in BRIEF working-memory scores.

## 4. Discussion

This study adds to the growing evidence of the importance of father–child RTP for child development. It expands upon previous findings by incorporating measures of working memory—one of the executive functions that underlies many aspects of everyday functioning. Furthermore, these findings add to the previous animal studies showing links between RTP and the PFC [15,17,18]. 

In support of our hypotheses, both RTP-Quality and RTP-Frequency were related to working-memory ability as measured by the WPPSI-IV. Furthermore, the regression model explained a very large amount of variance in working-memory ability (35%). Although this seems like an extremely high value, it is in line with a previous study whereby father–child toy play was found to explain approximately 25% of variance in cognitive development [48].

Our hypotheses around RTP and working-memory problems was partially supported in that RTP-Frequency, but not RTP-Quality, was associated with working-memory problems as measured by the BRIEF. Fathers who indicated they frequently engaged in RTP with the study child also rated that child as having fewer working-memory problems. This finding may be due to the self-report nature of each measure, or perhaps children with fewer working-memory problems enjoy engaging in RTP more frequently than those children who have difficulties planning and following instructions. Clearly this is an area that requires further investigation.

Interestingly, it was found that RTP-Quality and RTP-Frequency are not related to each other. This is important as it suggests that simply participating in RTP more often does not necessarily mean that the play is of a higher quality than when fathers and their children only participate in RTP occasionally. This may have implications for a child’s development if highly frequent RTP is of a low quality, whereby the father dominates the game and shows little warmth or positive regard towards their child. A systematic review exploring the developmental impacts of dyadic father–child play demonstrated that a father’s negative affect towards their child during play predicted poorer social and behavioural outcomes for children, such as lower sharing, peer ratings, and social acceptance [49]. Furthermore, negative effects have also been found for play interactions, whereby fathers, who frequently engage in RTP, are submissive and allow their child to dominate the play. This research has demonstrated that less-directive father play behaviour is associated with child physical aggression [50] and that less-dominant father play behaviour is associated with lower child emotional regulation [5]. Thus, these findings emphasise the need to differentiate between frequency and quality of play interactions, when assessing their respective impacts on child development.

### Limitations

No study is without limitations. We have presented a small exploratory study that has expanded previous work looking at father–child RTP and social and emotional outcomes to include working memory. Our sample size limited the examination of multiple predictive variables, however, it is larger than some previously published studies of RTP [6,11] and contains an approximately equal representation of gender in the child sample. Finally, and most importantly, this is a correlational study, which prevents any causal claims from being made.

## 5. Conclusions

Studies examining the positive effects of father–child RTP are critical in increasing the recognition of fathers as being influential in their children’s social, emotional, and cognitive development. This study has contributed to the field of child development by demonstrating strong links between father–child RTP and children’s working-memory ability. It has, additionally, accentuated the unique contribution of play quality, beyond merely play engagement. Future research should examine a broader range of cognitive executive functions and explore potential mediators between RTP and children’s social, emotional, and cognitive outcomes.

## Figures and Tables

**Table 1:** Descriptive statistics (mean and standard deviation or frequency) for scores on the WPPSI-V and BRIEF working-memory scales and quality and frequency of father–child rough-and-tumble play (RTP), separated by child gender.

	Boys (N = 14)	Girls (N = 16)	Total
Working Memory			
WPPSI-IV	99.00 (12.82)	105.44 (11.09)	102.43 (12.17)
BRIEF	50.93 (11.33)	51.38 (9.47)	51.17 (10.20)
RTP			
Quality	61.11 (14.47)	61.50 (11.78)	61.32 (12.87)
Frequency			
*Never*	1	0	1
*1–3 times a week*	7	12	19
*4–6 times a week*	4	4	8
*Every day*	2	0	2

**Table 2:** Results from the two stepwise linear regressions using RTP-Quality and RTP-Frequency to predict working-memory ability using the WPPSI-IV and working-memory problems using the BRIEF.

Model	R^2^_adj_	β	BF_10_
WPPSI-IV	0.35 **		
*RTP-Quality*		0.346 *	18.08
*RTP-Frequency*		0.486 **	4.46
BRIEF	0.15 *		
*RTP-Frequency*		−0.424 *	2.34

* *p* < 0.05, ** *p* < 0.01. β = standardised coefficient.

## Data Availability

The data presented in this study are available on request from the corresponding author.
